# Extraoral implants in irradiated pacients

**DOI:** 10.5935/1808-8694.20130033

**Published:** 2015-11-02

**Authors:** Joaquim Augusto Piras de Oliveira, Márcio Abrahão, Luciano Lauria Dib

**Affiliations:** a MSc in Sciences - DDS; b Full Professor of Head and Neck Surgery - Department of Otorhinolaryngology - Federal University of São Paulo - UNIFESP; c Full Professor of Semiology - Paulista University - UNIP. Federal University of São Paulo - UNIFESP

**Keywords:** osseointegration, prostheses and implants, radiotherapy

## Abstract

The aim of this study is to analyze the success of extraoral osseointegrated implants used to support and contain prosthesis designed to rehabilitate craniofacial deformities.

**Method:**

This study was based on the retrospective assessment of charts from 59 patients submitted to cancer surgery and who received 164 extraoral implants to contain facial prosthesis.

**Results:**

Among 164 implants, 42 were fixed in previously irradiated regions. Eight of the implants did not have osseointegration; and from these, 2 were fixed in irradiated bone. The result show 116 (95.1%) successfully osseointegrated implants in non-irradiated sites. The success rate among 42 implants fixed in previously irradiated bones was 40 (95.3%) osseointegrated implants.

**Conclusion:**

The use of extraoral craniofacial implants represents a safe and effective approach to treat facial deformities as a support for the rehabilitation prosthesis. Radiotherapy treatment does not prevent osseointegration.

## INTRODUCTION

In the past, materials found in nature, such as clay, wood, horn and animal skin, gold and silver, were all used in attempts to restore facial loss of substance. After the industrial revolution, in mid-18^th^ century, new materials started to be created and some, such as paper, paraffin, plastics, acrylic and finally, silicone, started to be utilized in the prosthetic reconstruction of facial mutilations^1^. The prosthesis were glued to the face or supported in eyeglasses frames.

Before 1969, time when Bränemark's papers were published, there were attempts to use implants crossing the skin and fixing to bones - yielding very negative results -they were rejected by infection within a maximum time of 3 months^2^.

The clinical need of permanent percutaneous links is found not only to retain the prostheses, but it is also used in nephrology, cardiology, neurology, urology, otorhinolaryngology, orthopedic surgery, plastic reconstructive surgery, and in many other clinical disciplines. This clearly depicts the universal need to develop and establish a permanent percutaneous anchor^3^.

This opportunity came up in 1965, with the studies from Prof. P. I. Brånemark, who was unable to remove from the bony structure of dogs a titanium capsule used to support a magnification lens used to observe blood circulation. Apparently, such capsule made a single bond with the bone. Soon after, Brånemark started a new line of research, which led to the revolutionary concepts of osseointegration, which spread to different fields of medicine and dentistry. Research in dentistry started in 1965, and it was published in 1969 on osseointegrated titanium implants, aimed at replacing dental elements and allowed for a stable fixation on the bony structures of the maxillary teeth, crossing the gum. In 1977, after following clinical trials for 12 years, the osseointegration concept was accepted by medical authorities in Sweden. In this same year, the concept was extrapolated to other regions of the face, with the implant crossing the skin and enabling an excellent method to anchor auditory devices and facial prostheses. The first clinical case was done in a hearing loss individual, used to anchor an external hearing aid. In 1979, a second case served as anchor for the prosthetic ear of and individual who had lost his ear pinna because of a tumor ressection^4^, ^5^. In 1995, the concept of facial prosthesis anchoring was also accepted by the American FDA (*Food and Drug Administration)*.

However, with the advent of osseointegration, a number of limitations to its use cropped up during the first years of its applications. Individuals with diabetes mellitus, osteoporosis and, especially, irradiated patients, started to be advised against the implant. Radiotherapy was originally considered a contraindication to installing osseointegrated dental implants, as per published in the 1988 consensus^6^. Concerned with the secondary effects that radiation causes to the maxillary teeth in doses higher than 55 Gy, especially osteonecrosis, the same concern was automatically transferred to the extraoral implants^7^.

Although the risk of osteoradionecrosis contraindi-cates the use of implants as a means of support treatment for prosthesis, the benefit they bring about for patient rehabilitation is huge and cannot be downplayed. Of the losses in irradiated patients, the craniofacial regions are the most affected: frontal bone 50%; zygoma 20%; temporal bone 8%^8^.

The poor situation of the bony structure, usually modified by irradiation effects, may difficult osseointegration. The minimum trauma caused during bone perforation to place the implant can be a triggering factor for the onset of osteoradionecrosis, when carried out near the radiotherapy sessions. Notwithstanding, these effects may be overcome by increasing the contact of the bony structures with the surface implants. Originally, the extraoral implant had a smooth and ground surface, which along time was modified by the need to increase contact between the bone and the implant. These changes were based on acid blasting, anodization, implant design changes, with the aim of enlarging its external area. The radiotherapy effect and the osteoporosis in elderly patients are mentioned as the main causes of implant failure. The study of 631 implants installed on 107 irradiated individuals, within a 25-year period, compared to a control group, showed that implant failure rates are higher after previous radiotherapy. High failure rates happen after high doses of radiotherapy, or a long time after the irradiation. The cranial regions most affected by radiotherapy were the frontal bone, the zygoma, mandible and maxilla. The lowest failure rates were found in the maxilla^9^.

Notwithstanding, hyperbaric oxygenation (HBO) is advised in order to avoid post-radiotherapy osteoradione-crosis^10^. Studies carried out in the University of Götemburg showed that hyperbaric oxygen increases angiogenesis and metabolism, acting as a growth factor and bone tissue renovation. From the clinical standpoint, HBO enables a better implant osseointegration in irradiated bones; protection against osteoradionecrosis; surgical complication reduction and healing increase in irradiated tissues.

In a case review study which happened before 1968, there were osteoradionecrosis rates (ORN) of 11.8%, compared to 5.4% after 1968. Such difference is associated with the fact that many radiotherapy units exchanged their orthovoltage devices for megavoltage and supervoltage in this period. Summaries from this last decade point to a 2.1% of osteoradionecrosis rate in previously irradiated patients. At the same time, dentists and radiotherapies became more aware as to radiotherapy secondary risks, improving mouth care and avoiding adrenalin injection in post-radiotherapy surgeries^11^.

Irradiated tissues develop a tissue hypovascular-hypocellular-hypoxia which does not spontaneously revascularize. The results from Marx et al.^12^ showed that angiogenesis after normobaric oxygenation was a complete failure, suggesting that in this case, oxygen is a drug which requires hyperbaric pressure in order to generate therapeutic effects on hypovascular irradiated tissues. The indicated level is 2.4 atmospheres (ATA). The simple increase of available oxygen to the cell tissue does not seem, by itself, to stimulate angiogenesis. The stimulus seems to stem more from the pressure to which oxygen is submitted. The angiogenesis stimulus is mediated by macrophages which migrate and secrete a variety of biochemical messages, including a chemotactic angiogenesis factor in response to high levels of lactose existing within the wound spaces.

Osteoradionecrosis is biologically considered a complex of dead cells and the cellular functional reduction caused by the transfer of radiation energy. One study encompassing 536 patients enabled to analyze and classify three pathophysiological conditions for osteoradionecrosis: induced by initial trauma; induced by late trauma; and spontaneous. The results enabled to establish procedures recommended to delay radiation for 21 days after tissue damage; one relative contraindication to damage the tissues during the course of radiotherapy; one recommendation for using hyperbaric oxygen before the tissue damage; and one strong recommendation to promote the cleaning of irradiated patients. The time interval between the irradiation and the implant surgery has been considered important for graft survival.

The ideal time has not been yet established. From the viewpoint of the individuals, immediate rehabilitation is important and, therefore, the shorter the time between irradiation and implant surgery the better. From the viewpoint of tumor biology, one must wait between 1 and 3 years after tumor surgery to perform the implant surgery. From the radiobiological viewpoint, the optimum time will be when the tissue reactions after radiotherapy have declined and the healing phase is established, these are 2 to 4 months after radiotherapy. When we consider the risk reduction by trauma on the irradiated tissues, the ideal time would be 6 months to 1.5 years after the irradiation^13^.

In Brazil, extraoral implants started to be employed as an element of prosthesis support in the rehabilitation of people with facial mutilation as of 1995. The implants utilized at the time were imported and represented a high rehabilitation cost.

In this study we analyzed 59 individuals mutilated by surgery to treat malignant tumors, who received extraoral implants to support facial prostheses. Our main goal was to know the success rate of implants fixed on irradiated bones.

## METHOD

This study was approved by the Ethics in Research Committee on April 9, 2010 - case number 0339/10.

We took the following information from the retrospective analysis of the charts from 59 individuals implanted and rehabilitated: patient identification, institution where the procedure was carried out, age, gender, comorbidities, reason for the deformity, deformity site, surgical treatment, radiotherapy treatment, pre-implant grafting, pre-implant hyperbaric oxygenation (HBO), and osseointegration success rate.

As for inclusion criterion, we considered all the charts or clinical files from individuals treated for malignant tumors and submitted to the extraoral implant fixation procedure, carried out by the team between 1995 and 2010.

The data studied was plotted in an Excel 201 for Windows for proper information storage. The statistical analyses were done by the *Statistical Package for the Social Sciences* (SPSS) version 19.0 software for Windows and the R-Program version 2.11.0.

For the quantitative variables (numeric) we calculated some summary measures, such as mean, median, minimum and maximum values, standard deviation. The qualitative variables (categorical) were analyzed by means of calculating the absolute and relative (percentage) frequencies.

The inferential analyses employed with the goal of confirming or refuting the evidence found in the descriptive analysis were:

• Fisher's Exact test and its extension to study the association between the final assessment (success and failure); radiotherapy treatment before implant installation (yes/no); dose utilized in the radiotherapy treatment (50 Gy, 60 Gy).
• Mann-Whitney test (Siegel, 2006) in the comparison of the final assessment (success and failure) of the implants.
• Implant and prosthesis survival analysis is represented by the Kaplan-Meyer curve.

## RESULTS

The 59 individuals who were part of this study were implanted by the same team of professionals.

For analysis purposes, we considered the 59 cases of tumor resection, which cell types found were 35 (59.3%) squamous cell carcinoma; 15 (25.4%) basal cell carcinoma; two (3.4%) melanoma; one (1.7%) mucoepidermoid carcinoma; one (1.7%) basal-squamous cell carcinoma; one (1.7%) hemangioma; one (1.7%) rhabdomyosarcoma; one (1.7%), retinoblastoma; one (1.7%), adenocarcinoma and one (1.7%) schwannoma.

None of the individuals were submitted to hyperbaric oxygen (HBO).

Four (6.7%) individuals were submitted to chemotherapy; one (1.7%) before implant fixation and three afterwards (5.0%).

Of the 59 cancer-treated individuals, 14 (23.7%) were irradiated: 7 with 50 Gy and the other 7 with 60 Gy. Forty-two implants were installed in these 14 previously irradiated individuals. Eight (19.0%) were in the ear; 34(81.0%) were in the orbit. None in the nasal region. Of these 42 implants, only two (4.7%) did not have osseointegration (Table 1). The success rate was 95.3% of osseointegrated implants.

**Table 1:** Irradiated individuals and rate of osseointegration success.

Anatomical region	Number of individuals	Age (years) Mean (min-max)	Gender M/F	Number of installed implants	Number of lost implants	% of osseointegration success
Year	3	67.3 (52-76)	2/1	8	0	100.0%
Nasal	0	0	0	0	0	0
Orbital	11	53.4 (20-75)	7/4	34	2	94.1%
Total	14	56.4 (20-76)	9/5	42	2	95.3%

Forty-five (76.3%) of the individuals were not irradiated (Table 2).

**Table 2:** Individuals with cancer who were not irradiated and rate of osseointegration success.

Anatomical region	Number of individuals	Age (years) Mean (min-max)	Gender M/F	Number of installed implants	Number of lost implants	Rate of osseointegration success
Year	1	43,0(43-43)	0/1	3	0	100.0%
Nasal	10	65,0(27-82)	7/3	16	2	87,5%
Orbital	34	64,4(25-90)	25/9	103	4	96,2%
Total	45	66,8(27-85)	32/13	122	6	95,1%

**Figure 1:**
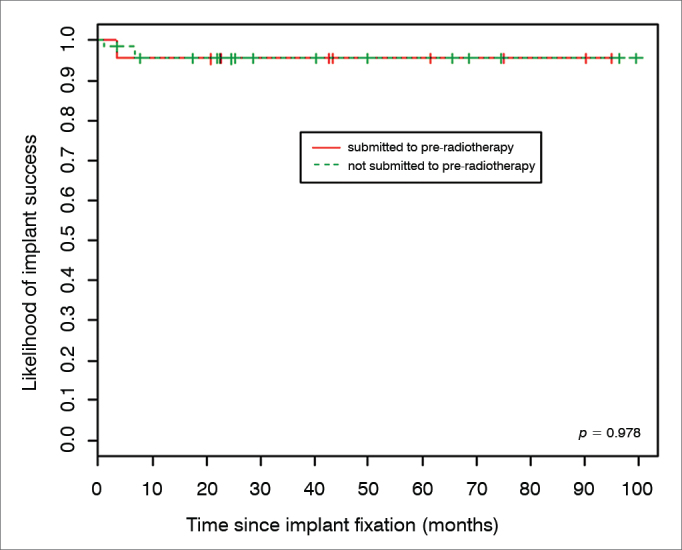
Rate of implant success according to radiotherapy.

Inferential results revealed that the expected likelihood of implant success is not associated to the pre-radiotherapy treatment (*p* = 0.978). According to Figure 1, the success estimated curves for individuals submitted to radiotherapy compared to those who were not submitted to radiotherapy have a very similar behavior.

## DISCUSSION

With osseointegration, special attention started to be paid to individuals who had been mutilated by cancer surgery, considering the possibility of obtaining a rigid Anchorage to support and retain facial prostheses.

Of the 59 individuals in this study, 41 were males and 18 were females.

The 59 cancer-treated individuals were considered in this study because of the possible need of having been treated with radiotherapy as coadjuvant to the surgical treatment. Fourteen (21.2%) of the individuals studied were submitted to radiotherapy before implant installation, in doses varying between 50 Gy for seven (50%) individuals and 60 Gy for seven other (50%) individuals.

Although HBO is an important factor to guarantee a greater likelihood of osseointegration in irradiated individuals, being reported as a stimulus to angiogenesis and metabolism, acting as bone tissue renovation and growth factor, preventing osteoradionecrosis^11^, ^12^, none of the individuals were submitted to HBO. Its use has been difficult in our country, because of the limited number of hyperbaric chambers available and its high cost. Of the 42 implants placed in 14 irradiated individuals, only two did not osseointegrated, resulting in a 95.3% success rate (Table 1). Of 122 implants placed in 45 non-irradiated individuals, only six did not have osseointegration, resulting in the 95.1% success rate (Table 2). These indices are very close, as per show non Figure 1.

These results are very coherent with other results published in the literature^14^, ^15^, ^16^, ^17^. And such values are considered much better than the 10% of losses recorded in the first 20 years of extraoral implant dentistry. We may consider that there has really been much development in implant dentistry in irradiated individuals as pertaining to surgical care, a proper post-surgical time to perform the implant, type of implant surface, hygienic care, following the radiation dose, besides the development of radiotherapy devices, which went from orthovoltage to supervoltage.

## CONCLUSION

Implants placed on previously irradiated surfaces have the same success rate as those placed on non-irradiated bone surfaces.
